# Olfactory training reduces pain sensitivity in children and adolescents with primary headaches

**DOI:** 10.3389/fpain.2023.1091984

**Published:** 2023-02-13

**Authors:** Gudrun Gossrau, Laura Zaranek, Anna Klimova, Rainer Sabatowski, Thea Koch, Matthias Richter, Antje Haehner

**Affiliations:** ^1^ Comprehensive Pain Center, University Hospital and Faculty of Medicine Carl Gustav Carus, TU Dresden, Dresden, Germany; ^2^ Department of Pediatrics, University Hospital and Faculty of Medicine Carl Gustav Carus, TU Dresden, Dresden, Germany; ^3^ NCT Partner Site Dresden, Institute for Medical Informatics and Biometrics, Faculty of Medicine Carl Gustav Carus, TU Dresden, Dresden, Germany; ^4^ Departement of Anesthesiology and Intensive Care Medicine, University Hospital and Faculty of Medicine Carl Gustav Carus, TU Dresden, Dresden, Germany; ^5^ Smell & Taste Clinic, Department of Otorhinolaryngology, TU Dresden, Dresden, Germany

**Keywords:** olfactory training, primary headache, pediatric migraine, smell training, pediatric headache

## Abstract

**Objective:**

Headache prevalence among children and adolescents has increased over the last few years. Evidence-based treatment options for pediatric headaches remain limited. Research suggests a positive influence of odors on pain and mood. We investigated the effect of repeated exposure to odors on pain perception, headache-related disability, and olfactory function in children and adolescents with primary headaches.

**Methods:**

Eighty patients with migraine or tension-type headache (mean 13.1 ± 3.29 years) participated, of whom 40 underwent daily olfactory training with individually selected pleasant odors for 3 months and 40 received state-of-the-art outpatient therapy as a control group. At baseline and after a 3-month follow-up, olfactory function [odor threshold; odor discrimination; odor identification; comprehensive Threshold, Discrimination, Identification (TDI) score], mechanical detection and pain threshold (quantitative sensory testing), electrical pain threshold, patient-reported outcomes on headache-related disability [Pediatric Migraine Disability Assessment (PedMIDAS)], pain disability [Pediatric Pain Disability Index (P-PDI)], and headache frequency were assessed.

**Results:**

Training with odors significantly increased the electrical pain threshold compared to the control group (*U* = 470.000; *z* = −3.177; *p* = 0.001). Additionally, olfactory training significantly increased the olfactory function (TDI score [*t*(39) = −2.851; *p* = 0.007], in particular, olfactory threshold, compared to controls (*U* = 530.500; *z* = −2.647; *p* = 0.008). Headache frequency, PedMIDAS, and P-PDI decreased significantly in both groups without a group difference.

**Conclusions:**

Exposure to odors has a positive effect on olfactory function and pain threshold in children and adolescents with primary headaches. Increased electrical pain thresholds might reduce sensitization for pain in patients with frequent headaches. The additional favorable effect on headache disability without relevant side effects underlines the potential of olfactory training as valuable nonpharmacological therapy in pediatric headaches.

## Introduction

Headache in children and adolescents is highly prevalent around the globe (1). Pediatric emergency departments count increasing visits and hospital admissions of children and adolescents with headaches (2). This might point to deficits in outpatient management. In addition, increasing headache frequency resulting from epigenetic and environmental changes has already been shown in a long-term cohort study from Finland. In a period of 30 years, a rise in migraine and tension-type headaches (TTHs) in both girls and boys could be detected (3).

Globally, headache disorders are known to be the second leading cause of years lived with disability (YLDs) in adults (4). In children and adolescents, data based on the Global Burden of Disease (GBD) study show that headaches account for 72% of all YLDs associated with neurological disorders (5). Between 2007 and 2017, YLDs associated with headache disorders increased (5).

Treatment methods for headaches in children and adolescents are limited. Nonpharmacological therapies such as educational programs or behavioral therapies are proven to have good effects (6, 7). However, their availability in everyday clinical practice is limited.

In adolescents with migraine, increased pain sensitivity has been detected (8). In children with migraine, a higher prevalence of sensory processing deviations resulting in sensory hypersensitivity has been shown (9). In this patient group, sensory processing difficulties correlated with lower quality of life. In adolescents with episodic migraine, avoidance of sensory input predicted the migraine-related disability level (10). Compared to those in patients with chronic migraine, pain thresholds are lower than in patients with episodic migraine, suggesting a link between pain sensitization and headache chronification.

Evidence-based specific pharmacological approaches are widely missing (11). This is not least due to the high placebo rate in children and adolescents, and large, high-quality studies could not show superiority for pharmacological migraine prophylaxis over placebo (12). Importantly, active pharmacological treatments show more side effects, which in turn lead to restrictions in daily life, compared to placebo. Response trajectories for therapy of children and adolescents with headaches show quick improvements (13). This positive aspect regarding the therapy of young headache patients should be taken into account in treatment. Overall, there is an unmet need to improve care at the primary, secondary, and tertiary levels. Especially new nonpharmacological treatment approaches with high therapeutic safety and wide availability also need to be further investigated and monitored for efficacy in everyday treatment. The aim is to reduce the disability of children and adolescents with headache disorders.

Among the nonpharmacological therapeutic measures of pain relief, odors have increasingly gained more attention over the last few years. In ear, nose, and throat (ENT), structured training with odors is already an acknowledged therapy in patients with smell loss. Previous research also suggests positive influences of training with odors on central nervous functions such as mood, cognition, and sleep (14–16). A study on patients with chronic low back pain using regular exposure to odors increased pain thresholds, suggesting that olfactory training might act by pain desensitization, which could be of value in reducing susceptibility to headaches and migraine attacks and their chronification (17). Crosstalk between the olfactory network and structures involved in the pain network, such as the insular cortex, cingulate gyrus, and hippocampus, has been hypothesized as a biological mechanism behind these effects. However, psychological mechanisms might play a role as well (18). Scientific evidence for the efficacy of psychological interventions in pediatric headaches is growing, and established nonpharmacological therapies comprise cognitive behavioral therapy, mindfulness meditation, and relaxation techniques, among others (19). In this clinical trial, we investigated the effect of training with odors on pain perception in children and adolescents with migraine or tension-type headaches and further assessed whether olfactory training affects the olfactory function and headache-related disability of these patients.

## Methods

The study protocol was approved by the Ethics Board of TU Dresden (protocol number EK-Nr. 386112011). Detailed information about the study was given to all participants and their parents and informed written consent was obtained. The conduct of the experiments complied with the World Medical Association Declaration of Helsinki. To detect differences in the olfactory function of the two groups with a medium effect size, a power of 0.8 and an alpha of 0.05, a sample size of 37 participants per group was chosen, and for a better assessment, 40 participants per group were defined.

### Study population

All participants were patients of a specialized pediatric headache outpatient clinic at the Pain Center of the University Hospital Dresden and received “state-of-the-art” treatment. During their appointment at the clinic, patients were approached for participation in the study; 40 patients were successively enrolled in an olfactory training group in the order of their treatment date, and the following 40 were enrolled in a control group with a state-of-the-art outpatient therapy. After 3 months, a follow-up was carried out. Exclusion criteria were age below 6 years, olfactory dysfunction, secondary headache disorders, or study rejection. All patients received a primary headache diagnosis according to the International Classification of Headache Disorders III (20).

### Psychophysical tests

Mechanical detection and pain thresholds and electrical detection and pain thresholds were obtained at baseline and after 3 months of olfactory training. Testing areas were the volar lower arms. The mechanical detection threshold (MDT) was measured with a standardized set of modified von Frey hairs that exert forces between 0.25 and 512 mN (21). Mechanical pain threshold (MPT) was measured using the PinPrick stimulators, which exert forces between 8 and 512 mN (21). For both tests, the geometric mean of five series of ascending and descending stimulus intensities was selected as the threshold value. For the electrical detection and pain thresholds, transcutaneous electrical nerve stimulation (TENS) equipment was used. TENS is used in a therapeutic setting, so it is a well-tolerated system to measure electric thresholds in children and adolescents (22). The electrical detection threshold was measured by a single stimulus of increasing electric current until participants detected the stimulus. After that, a stepwise increase in mA led to the level of perception of pain.

Olfactory testing was performed before and after the 3-month training period using the “Sniffin' Sticks” test kit (23), which involves tests for odor threshold, odor discrimination, and odor identification. The threshold test comprises 16 triplets of Sniffin' Stick pens, where one of the three pens is impregnated with N-butanol or phenylethylalcohol (BUT/PEA) diluted in a solvent according to a decreasing concentration. The children should specify the odor pen among the set of three pens presented. The second subtest assessed the ability of the patients to discriminate different odors. In this test, patients were also exposed to 16 triplets of odors, including two identical odors and one different odor. The task was to identify the odor, which differed from the other two pens. Eyes must be closed or blindfolded for both threshold and discrimination tests. The identification subtest consists of 16 common odors. The study participants were asked to choose from a list of four written proposals (24). The sum of the scores of the three subtests resulted in the Threshold, Discrimination, Identification (TDI) score, with a maximum of 48 points.

### Questionnaires

After psychophysical testing, each participant completed the following questionnaires: Pediatric Migraine Disability Assessment (PedMIDAS) to evaluate migraine-related disability (25), a pediatric headache questionnaire about headache medication, sports activities, mobile phone, or computer use (1), and Pediatric Pain Disability Index (P-PDI) (26). All questionnaires were completed at baseline and follow-up appointments.

### Training with odors

Olfactory training was carried out over 3 months with twice daily exposure to three pleasant odors, which were chosen at baseline. Eleven different odors were prepared for the selection of the three most pleasant (rose, orange, peach, lavender, lemon, cinnamon, apple, strawberry, chocolate, caramel, and clove). Patients were asked to sniff the odors for about 10 s each, according to the standard protocol for olfactory training based on a large number of studies (23, 27). To focus their attention on training, subjects were asked to keep a diary in which they rated their overall olfactory ability once a week. Children who felt hypersensitive to odors did not participate in the study. One participant dropped out of the study because the smell training induced migraine.

### Control condition

Patients in the control group received state-of-the-art outpatient treatment in the specialized children's headache outpatient clinic, like the intervention group. State-of-the-art outpatient treatment consists of regular, 3-monthly outpatient visits over 30 min. Individual education on the headache situation, prescription of structured applied relaxation techniques, physiotherapeutic treatments, and outpatient behavioral therapy were given according to individual needs. Acute drug therapy was prescribed, and often nutritional supplements such as magnesium were advised. Pharmacologic headache prophylaxis was rarely used in the case of refractory headaches.

### Statistical analyses

Statistical analysis of the data was conducted using IBM SPSS Statistics Version 21 (SPSS Inc., Chicago, IL, United States). Normal distribution was tested with the Shapiro–Wilk test.

Depending on whether or not a significant deviation from normality was found, further investigation of group differences was carried out using either the Mann–Whitney *U*-test and Wilcoxon test or a *t*-test (two-sample independent or dependent). Data are summarized as relevant in terms of mean ± SD, percentage, or standardized ratio.

The significance level was set as *p* < 0.05 (two-tailed test). In addition, for each outcome of interest, we fitted a linear mixed-effects regression model with the main and interaction effects for group and time, as well as a random intercept per subject, and reported the fit results, in particular, the *F*-statistics with a corresponding *p*-value, and *η*², as a measure of the effect size. However, because only a single follow-up measurement per patient was taken, the chance of confounding between the group and time effects could be very high. We, therefore, suggested drawing main conclusions from the univariate, parametric, or nonparametric analyses of relative differences toward the baseline. To carry out the multivariable analyses for outcomes of interest, adjusting for relevant covariates, the mixed-effects regression model was employed.

Spearman correlation analysis was conducted to investigate the relationship between a change in smell perception and changes in sensory thresholds, headache frequency, and disability. The change of either value has been calculated as a ratio: [(value_3 months_ – value_baseline_)/value_baseline_].

## Results

Eighty children and adolescents with primary headache disorders [51 females, 29 males, mean age (range) = 13.1 (6–19) years] participated in the study. The mean age in the olfactory training group was 13.83 years and in the control group was 12.38 years (*p* = 0.048). In the olfactory training group, 60% of the participants were females, and in the control group, 67.5% were females.

Out of the 80 participants in this study, 24% were diagnosed with migraine without aura, 16% with migraine with aura, 21% with TTH, and 39% with both migraine and TTH (also named as mixed headache). In the olfactory training group, most participants had the diagnosis of migraine without aura (12 participants, 30%) and mixed headache (14 participants, 35%). In the control group, most of the participants had a mixed headache diagnosis (17 participants, 43%) (Table 1).

**Table 1:** Demographic and clinical data of the olfactory training group and control group.

	Olfactory training group	Control group	Standardized mean difference
Mean age	13.83 (SD ± 3.18)	12.38 (SD ± 3.27)	0.45
Sex	60% female (28)	67.5% female (29)	−0.16
	40% male (17)	32.5% male (14)	0.16
Headache diagnosis	MwoA 30% (10)	MwoA 17.5% (11)	0.30
	MwA 12.5% (5)	MwA 20% (12)	−0.20
	TTH 22.5% (13)	TTH 20% (12)	0.06
	Mixed 35% (15)	Mixed 42.5% (20)	−0.15
Headache days (last 3 months) baseline/follow-up	30.5 (SD ± 27.1)	33.1 (SD ± 31.5)	−0.09
	24.8 (SD ± 27.6)	23.4 (SD ± 28.1)	0.05
PedMIDAS baseline/follow-up	31.1 (SD ± 25.3)	30.3 (SD ± 42.9)	0.02
	23.7 (SD ± 24.6)	19.1 (SD ± 23.1)	0.19
TDI score baseline/follow-up	38.5 (SD ± 4.2)	38.5 (SD ± 4.1)	0
	40.2 (SD ± 3.7)	39.1 (SD ± 3.0)	0.33

Standardized mean difference was used to depict effect sizes of group differences.

MwoA, migraine without aura; MwA, migraine with aura; TTH, tension-type headache; PedMIDAS, Pediatric Migraine Disability Assessment Score; TDI score, Threshold, Discrimination, Identification score.

About 35% of the participants had concomitant diseases. The most common other diagnoses were asthma (14%), endocrine disorders like hypothyroidism (7%), pain disorders like back pain (11%), and mental disorders like depression (11%) and anorexia (3.5%) (Table 2).

**Table 2:** Concomitant diseases in study participants.

Participants without concomitant diseases	Participants with concomitant diseases
*n* = 52/80 (65%)	*n* = 28/80 (35%)
**Concomitant disease**	
Allergic asthma	*n* = 4/28 (14%)
Hypothyroidism	*n* = 2 (7%)
Back pain	*n* = 3 (11%)
Depression	*n* = 3 (11%)
Anorexia	*n* = 1 (3.5%)

### Olfactory function

Baseline olfactory testing revealed normosmia in both treatment groups (Table 1) based on normative data in more than 9,000 children and adolescents (28).

**Figure 1:**
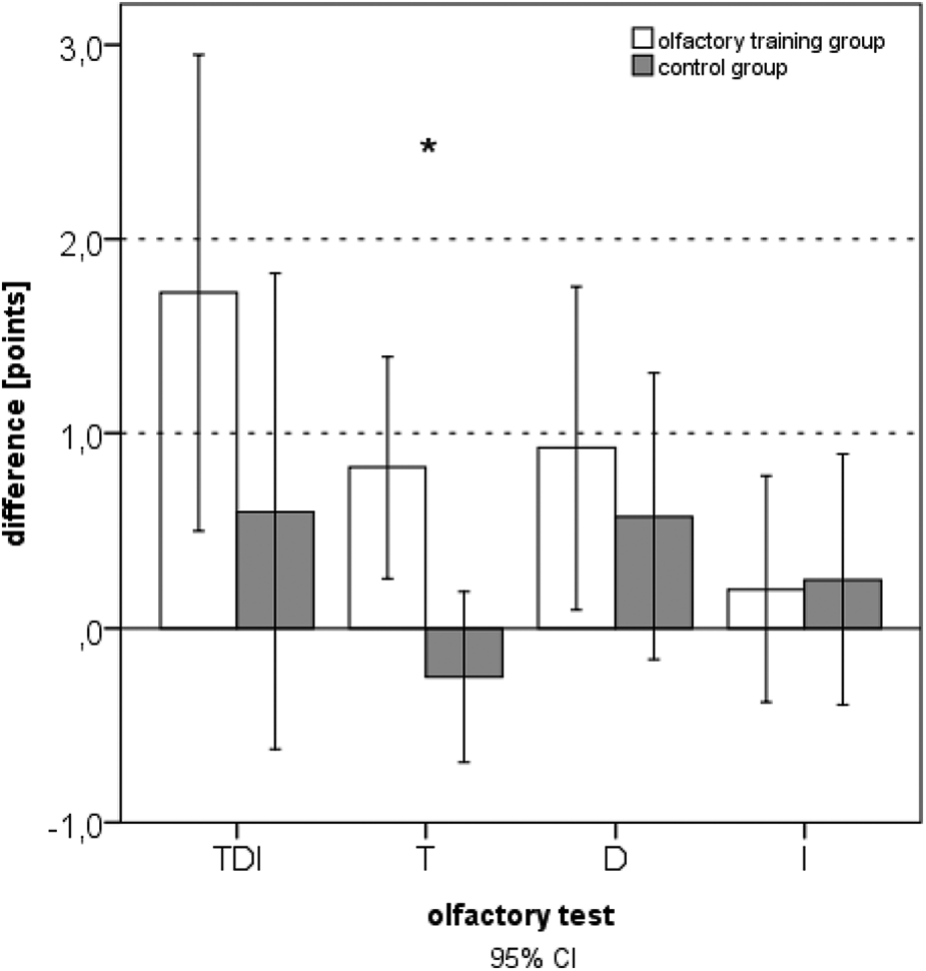
Difference in olfactory function after 3 months of training (expressed as comprehensive TDI scores and subtests: T, threshold; D, discrimination; I, identification).

After 3 months of odor exposition, a significant improvement in the olfactory function reflected by an increased TDI score was observed for the olfactory training group [*t*(39) = −2.851; *p* = 0.007]. Furthermore, the olfactory training group exhibited significantly improved odor discrimination [*t*(39) = −2.254; *p* = 0.030]. No significant changes in the olfactory function were observed for the control group. The change in olfactory thresholds was significantly different between the training and control groups (Mann–Whitney *U*-test: 540.500; *z*: −2.082; *p*: 0.01) (Figure 1). Gender had no statistical influence on TDI improvement [*F*(1,76) = 0.71; *p* = 0.40]. ANOVA also identified a significant main effect of olfactory training duration and TDI improvement [*F*(1,78) = 7.393; *p* = 0.008; part. *η*² = 0.087].

In the olfactory training group, the 3-month follow-up data indicated significantly increased TDI scores (Wilcoxon test: *z* = −2.354; *p* = 0.019; *n* = 40). There was no change in TDI in the control group after 3 months (Wilcoxon test: *z* = −0.37; *p* = 0.712; *n* = 40). Comparing the two groups at baseline, no statistical difference in the olfactory function was seen (*U* = 621.500; *z* = −1.718; *p* = 0.086). Comparing the two groups at follow-up after 3 months, a significant difference in the olfactory function was evident.

### Sensory thresholds

The MDT (mean olfactory training group at baseline: 0.46 mN; SD: ±0.62; mean olfactory training group at follow-up: 0.90 mN; SD: ±1.86; Wilcoxon test: *z* = −2.354; *p* = 0.019; *n* = 40) changed significantly in the olfactory training group. No change was seen in the control group (mean control group at baseline: 0.27 mN; SD: ±0.14; mean control group at follow-up: 0.27 mN; SD: ± 0.10; Wilcoxon test: *z* = −0.37; *p* = 0.712; *n* = 40). The olfactory training group showed significantly increased MDT values at the follow-up appointment compared to the control group (*U* = 444.00; *z* = −3.429; *p* = 0.001).

The MPT did not significantly differ in the olfactory training group (mean olfactory training group at baseline: 33.48 mN; SD: ±44.96; mean olfactory training group at follow-up: 40.82 mN, SD:±63.27; Wilcoxon test: *z* = −0.927; *p* = 0.354; *n* = 40) and control group (mean at baseline: 29.66 mN, SD: ± 31.77; mean at follow-up: 25.88 mN; SD: ± 27.11; Wilcoxon test: *z* = −1.371; *p* = 0.170; *n* = 40). The difference between the two groups did not significantly differ at follow-up (*U* = 634.500; *z* = −1.593; *p* = 0.11).

Correlation analysis explored a positive correlation between the change in olfactory function (TDI ratio) and the change in MPT ratio (*p* = 0.051) but no correlation between the change in olfactory function (TDI ratio) and the change in MDT.

### Electric perception and pain threshold

**Figure 2:**
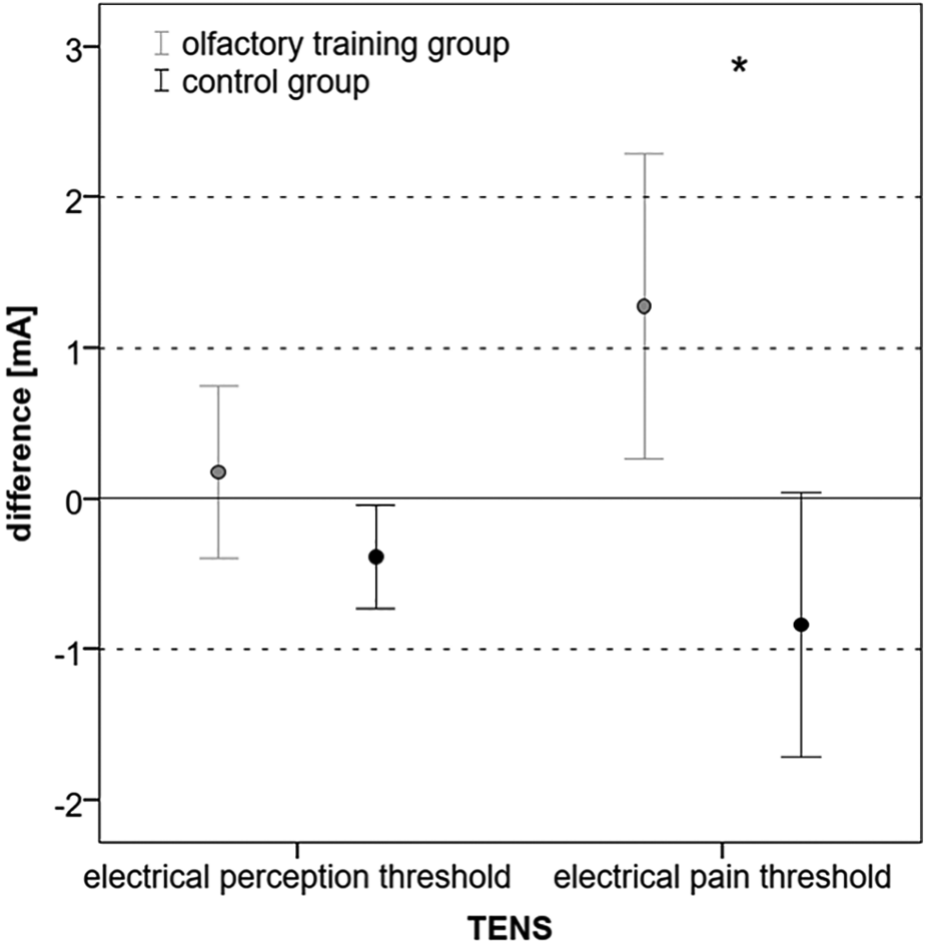
Difference (follow-up − baseline) in electrical perception and pain threshold in mA (*p < 0.05).

The electrical perception threshold did not differ in the olfactory training group (mean at baseline: 5.37 mA, SD: ± 1.38; mean at follow-up: 5.55 mA, SD: ±1.78; Wilcoxon test: z = −0.234; *p* = 0.815; *n* = 40) and control group (mean at baseline: 5.23 mA; SD: ±1.64; mean at follow-up: 4.85 mA; SD: ±1.65; Wilcoxon test: *z* = −1.883; *p* = 0.060; *n* = 40). The electrical pain threshold significantly changed for both groups after 3 months. In the olfactory training group, the electrical pain threshold increased significantly after the olfactory training (mean at baseline: 11.68 mA; SD: ±3.52; mean at follow-up: 12.96 mA; SD: ±4.99; Wilcoxon test: *z* = −2.217; *p* = 0.027; *n* = 40). In the control group, the electrical pain threshold significantly decreased after 3 months (mean at baseline: 12.33 mA, SD: ±3.97; mean at follow-up: 11.50 mA; SD: ±4.38; Wilcoxon test: *z* = −2.283; *p* = 0.022; *n* = 40). Differences between the two groups changed significantly (mean difference olfactory training group: 1.27 mA; SD: ±3.16; mean difference control group: −0.83 mA; SD: ±2.73; Mann–Whitney *U*-test: 493.000; *z*: −2.954; *p*: 0.003) (Figure 2). Based on ANOVA, the interaction effect between the study group and time on the electrical pain threshold was found significant [*F*(1,78) = 6.746; *p* = 0.011; part. *η*² = 0.080]. There was no statistical difference in headache diagnosis and electrical pain perception [chi-quadrat (2) = 3.562; *p* = 0.31].

Correlation analysis explored a positive correlation between the change in olfactory function (TDI ratio) and the change in electrical pain threshold (ratio: *p* = 0.021).

### Headache frequency and headache-related impairment

In the olfactory training group, headache frequency decreased from mean 30.5 to 25 days after 3 months of smell training (Wilcoxon test: *z* = −3.015; *p* = 0.003; *n* = 40). In line with it, the PedMIDAS score decreased in the olfactory training group after 3 months of olfactory training from mean 31.1 to 24.5 (Wilcoxon test: *z* = −2.183; *p* = 0.029; *n* = 40).

**Figure 3:**
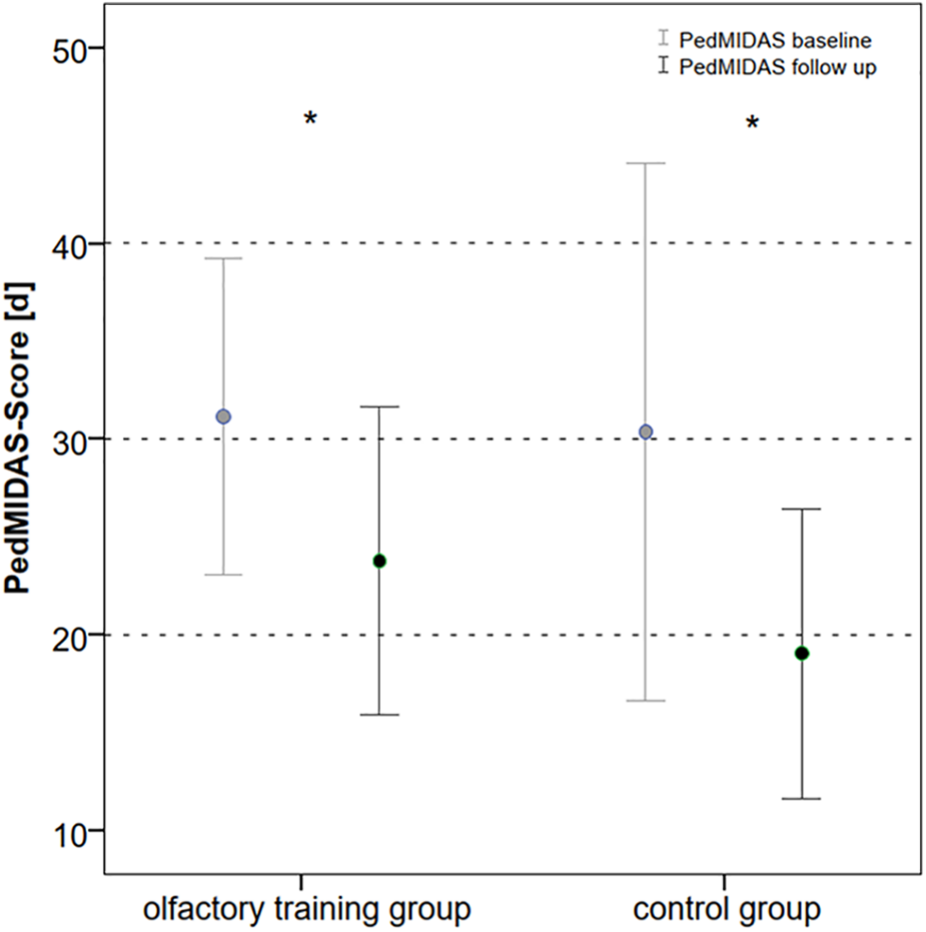
PedMIDAS score at baseline and follow-up in olfactory training group and control group (in days of disability within the last 3 months, *p < 0.05). PedMIDAS, Pediatric Migraine Disability Assessment.

The headache frequency in the control group was 33.1 days at baseline mean and decreased to 23.4 days at follow-up (Wilcoxon test: *z* = −2.162; *p* = 0.031; *n* = 40); the PedMIDAS score decreased from mean 30.3 points at baseline to 19.1 points at the follow-up appointment (Wilcoxon test: *z* = −2.098 *p* = 0.037, *n* = 40) (Figure 3). The comparison of the differences between the two groups showed no statistically significant difference in headache frequency (Mann–Whitney *U*-test:680.000; *z*: −0.807; *p*: 0.41) and PedMIDAS (Mann–Whitney *U*-test: 647.000; *z*: −1.134; *p*: 0.25). There were no significant differences in headache-related impairment with reference to the headache diagnoses. ANOVA showed a main effect on study time and headache frequency [*F*(1,78) = 13.081; *p* = 0,001; part. *η*² = 0.144] and PedMIDAS score [*F*(1,78) = 8.580; *p* = 0.004; part. *η*² = 0.099] but with no impact of the group. However, the main effect of the age group on headache frequency was shown [*F*(3,76) = 4.192; *p* = 0.008; part. *η*² = 0.142]. The age group from 6 to 9 years experienced the greatest reduction in headache days in both groups (Tables 3,4).

**Table 3:** Headache reduction (mean days) in different age groups at baseline and follow-up appointments—the olfactory training group and control group were pooled.

Age group (years)	Mean days in headache reduction
6–9	−21.8
10–12	−4.3
13–15	−7.7
16–19	−2.1

**Table 4:** Headache reduction (mean days) in different age groups at baseline and follow-up appointments—olfactory training group and control group.

	Olfactory training group				Control group			
	Age group (years)				Age group (years)			
	6–9	10–12	13–15	16–19	6–9	10–12	13–15	16–19
Headache days of last 3 months at baseline (m)	33	22	23	38	42	20	42	30
Headache days at follow-up (m)	19	17	20	33	16	17	29	32

m, mean.

Correlation analysis explored a significant negative correlation between the change in olfactory function (TDI ratio) and the change in headache days (ratio, *p* = 0.023). Interestingly, a negative correlation between the change in electrical pain threshold and the change in headache days (ratio, *p* = 0.053) and a significant negative correlation between the change in electrical pain threshold and the change in headache disability (PedMIDAS ratio, *p* = 0.011) were found.

### Medication intake

**Figure 4:**
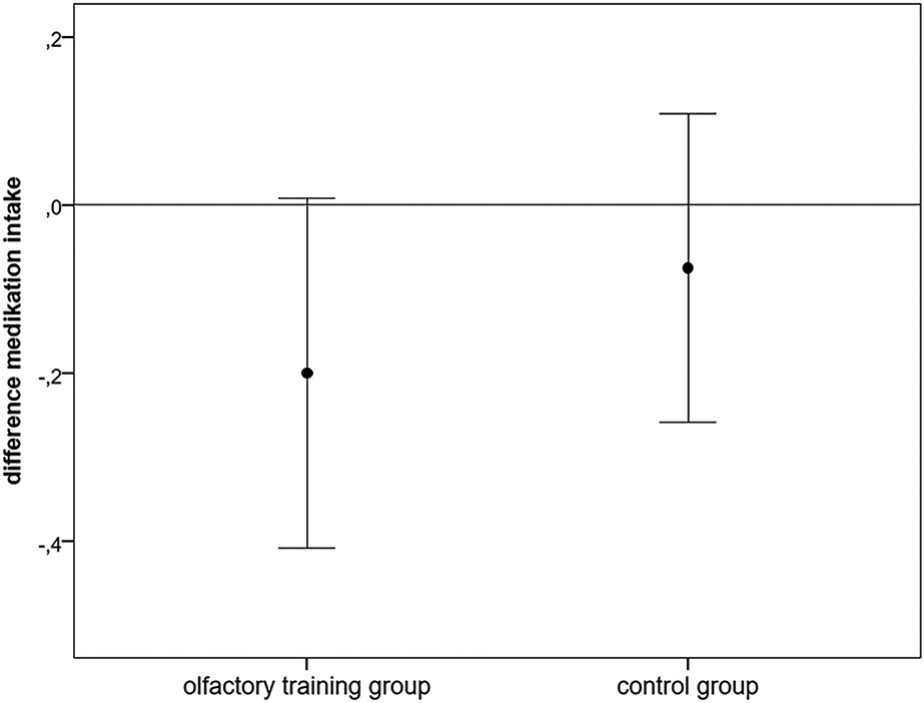
Difference (follow-up − baseline) in analgesic medication intake (points: 0: no medication intake, 1: ≥ once/months, 2: ≥once/week).

Medication intake for the last 3 months was inquired about by an individual headache questionnaire (1). Information about medication intake was categorized in the following points: 0 = no medication intake, 1 = medication intake at least once per month, and 2 = medication intake at least once per week. There was no significant difference in the ratio of medication intake between the two groups at baseline (Mann–Whitney *U*-test: 698.000; *z*: −1.253; *p*: 0.2). A marginal reduction in medication intake was found in the olfactory training group (Wilcoxon test: *z*: −1.84; *p*: 0.06) but not in the control group (Wilcoxon test: *z* = −0.832; *p* = 0.405; *n* = 40) (Figure 4).

The participants used ibuprofen (68%), acetaminophen (20%), dipyrone (1%), and triptane (11%) for pain medication at baseline, with no difference between the two groups (Supplementary Figure S1). At the follow-up appointment, the usage of ibuprofen was reduced to 61%, acetaminophen intake was increased to 25%, and triptane usage was increased to 14%. When comparing the ibuprofen intake between the two groups at baseline, 55% of the participants of the olfactory training group and 80% of the participants of the control group used ibuprofen. At follow-up, 50% of the participants of the olfactory training group and 62.5% of the participants of the control group used ibuprofen. The paracetamol intake increased from 15% at baseline to 20% at follow-up in the olfactory training group and from 25% at baseline to 30% at follow-up in the control group. Triptane usage in the olfactory training group was 17.5% at baseline and did not change at follow-up, compared to the control group, where triptane intake at baseline was 5% and increased to 10% at follow-up.

### Headache intensity/sleep/mood

Within the PedMIDAS score, headache intensity, sleep quality, and mood were patient-reported. There was no significant difference in the headache intensity ratio between the two groups (Mann–Whitney *U*-test: 685.500; *z*: −0.941; *p*: 0.347) (Supplementary Figure S2).

**Figure 5:**
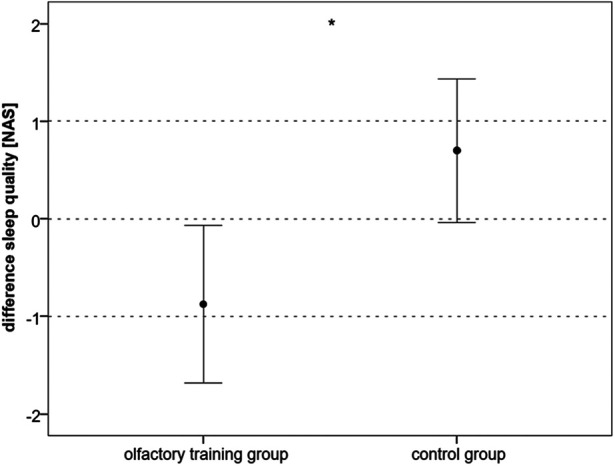
Difference (follow-up − baseline) in sleep quality, subscale of PedMIDAS (NAS: 1 very good sleep quality, up to 10: very bad sleep quality) (*p < 0.05). PedMIDAS, Pediatric Migraine Disability Assessment; NAS, numeric analog scale.

There was a significant improvement in sleep quality in the olfactory training group [mean at baseline: 4.08 points; SD ±2.73; mean at follow-up: 3.2 points; SD ±2.52; *t*(39) = 2.184; *p*: 0.035]. The sleep quality of the control group aggravated [mean at baseline: 3.4 points; SD ±2.52; mean at follow-up: 4.15 points; SD: ±2.66; *t*(39) = −1.924; *p*: 0.062]. There was a significant difference in the sleep quality ratio between the two groups (Mann–Whitney *U*-test: 464.500; *z*: −2.082; *p*: 0.03) (Figure 5). The olfactory training group had improved sleep quality.

In the olfactory training group, there was a significant improvement in mood quality after 3 months of olfactory training [mean at baseline: 4.03 points; SD ±2.08; mean at follow-up: 3.23 points; SD ±2.00; *t*(39) = 2.109; *p* = 0.041]. There was no significant change in the control group [mean at baseline: 4.03 points; SD ±2.03; mean at follow-up: 3.9; SD ±2.02; *t*(39) = 0.376; *p* = 0.71].

## Discussion

The results of this controlled prospective study suggest that structured olfactory training not only improves olfactory function but also increases the electrical pain threshold and significantly impacts the mechanical detection threshold in children and adolescents with primary headaches. Both training and control groups experienced a significant reduction in headache frequency and headache-related disability. Overall, patients up to 9 years improved more in headache frequency than older patients, especially adolescents of 16 years and older. In addition to participation in the study, both groups continued to receive continuous outpatient headache therapy in the form of prescription of acute medication, relaxation training, and motivation to engage in endurance sports. The consistent headache reduction in both groups might be due to both implementation of the already-known therapy content and a placebo effect. The differences described here could indicate a different response to outpatient therapies or a higher placebo response in the younger age group. Previous studies in children and adolescents on the effect of triptans in migraine therapy have shown that placebo levels in youth are significantly higher than in adults (30). However, studies tailored to examine concrete questions concerning age-dependent therapy and placebo responses are needed. A reduction in analgesic intake was found in the olfactory training group only. In addition, sleep and mood improved significantly in patients' self-reported scales, whereas headache intensity remained unchanged.

However, smell-induced migraine attacks can be a limiting factor in olfactory training. Exposition to odors in patients with primary headaches has shown that attacks were triggered in about one-third of migraine patients (31). Interestingly, odors triggered headaches in patients with migraine only but not in patients with other primary headache disorders. One patient dropped out of our study because olfactory training frequently induced migraine attacks.

Structured olfactory training is already established in the treatment of hyposmia (32). In addition, positive effects of odor training on mood and concentration in adults have been shown (33). For children and adolescents with primary headaches, restrictions in sleep quality are known, but also restrictions in affect and the ability to concentrate (29). In this context, the results of this study point the way to further research on improving these comorbidities through exposure to pleasant odors. In adults with chronic back pain, scent training did not change pain intensity but led to an increased pain threshold (17), which is in line with the results of the current study in children with recurrent headaches. Data in mice showed reduced activation of the spinal trigeminal nucleus caudalis after olfactory costimulation with floral odorants and the application of a trigeminal pain stimulus (34). However, this study refers to changes in the caudal nucleus of the trigeminal nerve after scent exposure. In the data shown here, changes in pain threshold occur in a noncephalic region, the volar forearm. Similar effects were shown in the study of back pain patients after training with positive scents. There, an increase in the electrical pain threshold was also shown in a noncephalic area (17), suggesting that changes in trigeminal processing affect pain perception beyond cephalic regions. In fact, animal work revealed that stimulation of upper body regions, including the meninges and brainstem, with IL-6 could cause widespread hypersensitivity in the whole body. On the contrary, IL-6 stimulation of the lower body did not induce hypersensitivity of cephalic regions (35). Previous data proved that sensitization of third-order neurons mediates cutaneous allodynia not only in the head but also in the forearm of humans (36). Because of consecutive sensitization of first-, second-, and eventually third-order neurons, it has been suggested to use acute antimigraine treatments early in the attack to avoid spreading the sensitization to the third-order neuron and following noncephalic allodynia with increased pain. Clinical implementation has been shown to be effective (36).

Based on the data shown here, we hypothesize that training with odors may counteract the process of sensitization of trigeminal second-order neurons, as expressed by the increased pain threshold. Thus, olfactory training may have the potential to help to counteract the vicious circle of sensitization that is underlying chronic headache and pain.

## Limitations

The most relevant limitation of this study is the missing placebo control. Overall, a selection bias exists for patients who regarded themselves as nonosmophobic. The age difference between intervention and control groups constitutes a limitation and is based on successive patient recruitment. Since placebo effects decrease with the increasing age of children, a slightly higher average age of the intervention group is not considered critical for the study results. However, the younger average age of the control group could lead to larger placebo effects and difficulties in proving specific effects of the olfactory training.

## Conclusion

Olfactory training is a well-tolerated and inexpensive nonpharmacological treatment option that might positively influence childhood headaches.

## Data Availability

The raw data supporting the conclusions of this article will be made available by the authors, without undue reservation.
