# Supplementary material for: Olfactory training reduces pain sensitivity in children and adolescents with primary headaches
Source: Front Pain Res (Lausanne). 2023 Feb 13;4:1091984. doi: 10.3389/fpain.2023.1091984 (PMC9968932; doi:10.3389/fpain.2023.1091984)

Supplementary Figures

Supplementary Figure 1.

Distribution of acute headache medication at baseline

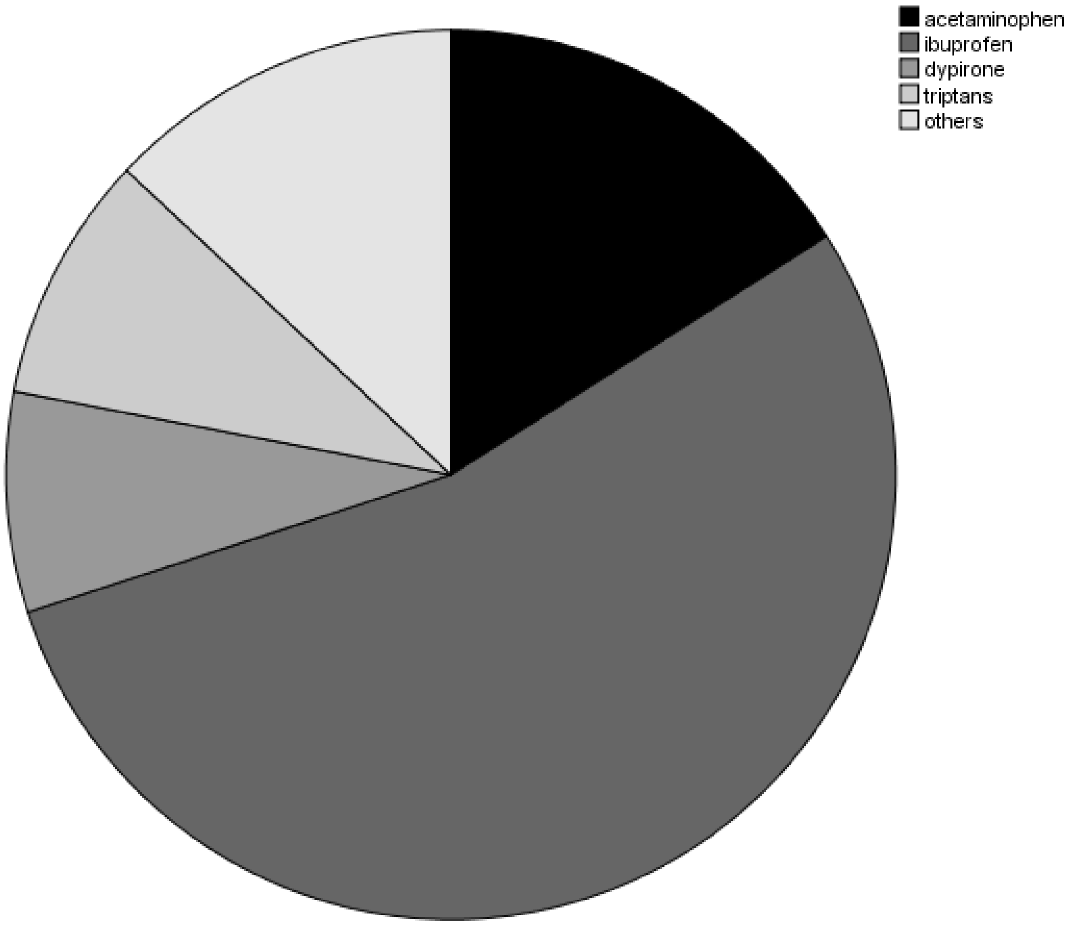

Supplementary Figure 2

Difference (follow up – baseline) in headache intensity, Subscale of PedMidas (numeric analoge scale: 1 low intensity, up to 10: very high intensity)

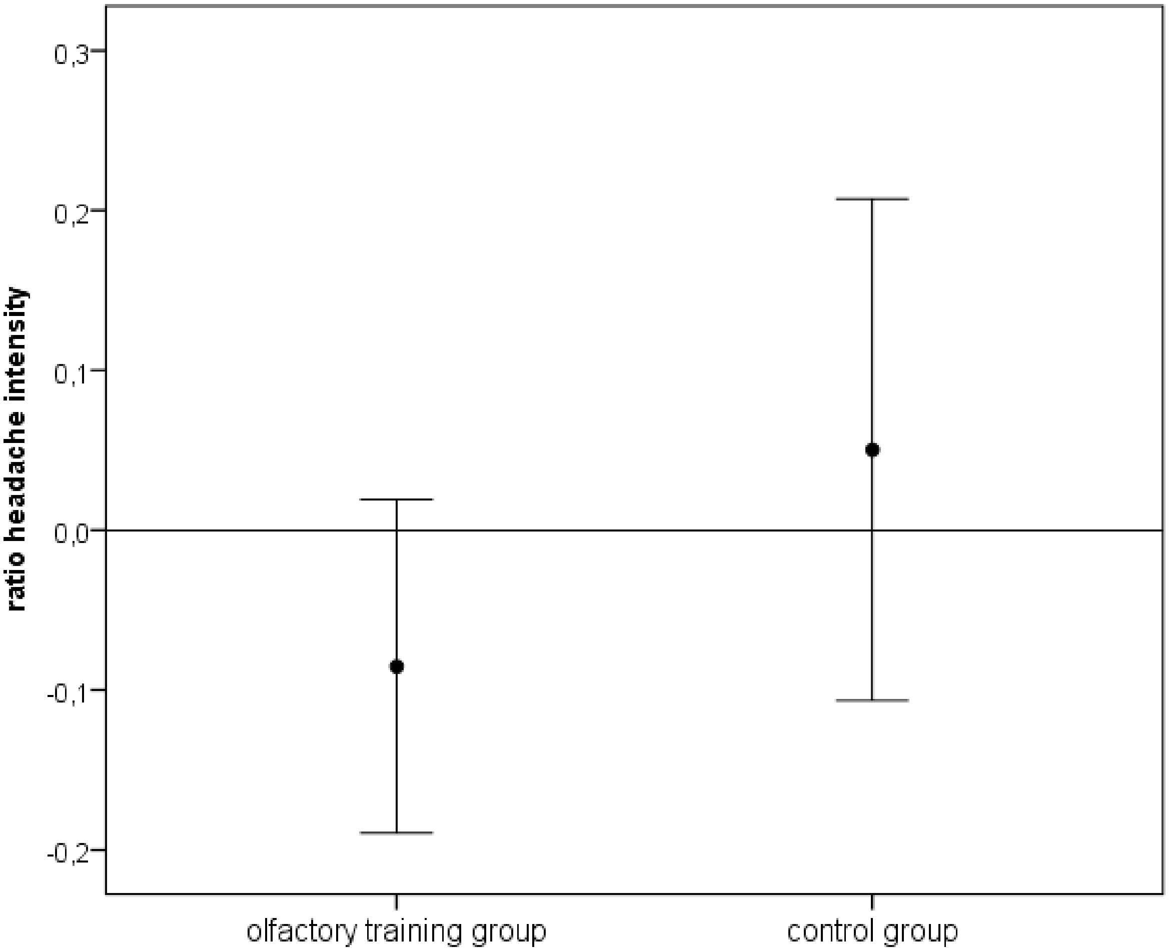

Supplement: Supplementary file 1 [file Datasheet1.pdf]
